# Psychological Intervention to Improve Communication and Patient Safety in Obstetrics: Examination of the Health Action Process Approach

**DOI:** 10.3389/fpsyg.2022.771626

**Published:** 2022-02-18

**Authors:** Christina Derksen, Lukas Kötting, Franziska Maria Keller, Martina Schmiedhofer, Sonia Lippke

**Affiliations:** ^1^Health Psychology and Behavioral Medicine, Psychology and Methods, Jacobs University Bremen, Bremen, Germany; ^2^Coalition for Patient Safety e.V., Berlin, Germany

**Keywords:** communication, obstetrics, healthcare workers (HCW), health action process approach (HAPA), patient safety

## Abstract

**Background:**

Human failure and a lack of effective communication are the main reasons for preventable adverse events, compromising patient safety in obstetrics. In order to improve safety, team and communication interventions have been implemented but lack feasibility in obstetric care. Psychological models such as the health action process approach might help to improve interventions.

**Methods:**

In a cross-sectional online survey with *N* = 129 healthcare workers (Study 1) and a paper-pencil survey with *N* = 137 obstetric healthcare workers at two obstetric university hospitals (Study 2), associations of social-cognitive variables were tested in a path analysis and a multiple regression. Preliminary results informed a communication training for all obstetric healthcare workers. A repeated-measures MANOVA was used to compare pre- and post-intervention data.

**Results:**

Social-cognitive variables were associated according to model suggestions (β = –0.26 to 0.45, *p* < 0.05) except for planning in the first study. Triggers of adverse events were associated (β = –0.41 to 0.24, *p* < 0.05) with communication behavior (Study 2), action self-efficacy and planning (Study 1), as well as barriers to effective communication (both studies). The intervention was rated positively (*M* = 3.3/4). Afterward, fewer triggers were reported and coping self-efficacy increased. There were group differences regarding hospital, experience, and time.

**Discussion:**

The health action process approach was examined in the context of safe communication in obstetrics and can be used to inform interventions. A theory-based, short training was feasible and acceptable. Perceived patient safety improved but communication behavior did not. Future research should aim to test a more comprehensive psychological communication intervention in a thorough RCT design.

## Introduction

In the last decades, research in health-related fields has increasingly focused on patient safety, defined by the absence of harmful incidents of patient harm that could have been avoided under given circumstances (Runciman et al., 2009). Although these adverse events are usually rare, they can lead to lasting disabilities and contribute to patient death, especially in emergency medicine (Pettker, 2017; Eulmesekian et al., 2020). A substantial portion of harmful events can be considered preventable when applying ordinary standards of care (Shojania and Marang-van de Mheen, 2020), and are therefore called “preventable adverse events” (pAE; Schrappe, 2018).

Preventable adverse events with severe consequences have been reported in gynecology and obstetrics (Zech et al., 2017). Adverse care processes can cause maternal and fetal adverse events that have been linked to trauma to newborns, delays in treatment resulting in worsening conditions, and parents’ emotional distress (Forster et al., 2006). In retrospective analyses, adverse care processes leading to neonatal death were commonly characterized by a lack of safe communication between doctors, nursing staff, and patients (Joint Commission, 2021). Nevertheless, a clear understanding of effective professional communication is yet to be achieved. Communication has been incorporated in multiple clinical care training programs. The “TeamStepps” program utilizes communication methods to ensure clear technical communication within teams, particularly in emergency medicine (Obenrader et al., 2019; Matzke et al., 2021). Recently, a review has concluded that it can improve communication, decrease clinical error rates, and improve patient satisfaction (Parker et al., 2019). However, the program focuses on different aspects of teamwork and performance instead of communication.

Major challenges in healthcare all over the world are changing working conditions, economic constraints, and increased patient-staff ratios (Institute of Medicine, 2000), leading to potentially unsafe care. Since HCW are working under time constraints interfering with their daily patient care, comprehensive training programs might not be acceptable and feasible. HCW need time and cognitive resources to pursue education and training at work while holding up an effective patient care, thus making more targeted training interventions necessary. A lack of training and preparation has been reported as a barrier to effective patient-centered care and communication (Paternotte et al., 2015), which applies to sexual and reproductive healthcare (Morris and Rushwan, 2015).

To develop a feasible but targeted training program, influences on team and patient-provider communication need to be examined. Relevant influences on team communication include organizational aspects such as team climate and work policies (González-Romá and Hernández, 2014) that can be classified into four categories: (a) clinical environment, including work overload; (b) interpersonal relationships and hierarchies; (c) personal factors such as self-efficacy; and (d) lack of training (Olde Bekkink et al., 2018). Healthcare teams are complex and dynamic with HCW coming from different professional and cultural backgrounds (Pype et al., 2018; Kaihlanen et al., 2019). Interpersonal relations are crucial for team communication and affect team performance and patient safety (Lee and Doran, 2017) but have not sufficiently been addressed in education and training. Future directions for policymakers include adding communication and patient safety education to curriculums as well as applying handover tools and simulation trainings (Foronda et al., 2016).

In patient-provider communication, similar barriers and facilitators have been found. HCW have emphasized the role of knowledge regarding care issues but are in need of developing skills and competencies to put their knowledge into practice (Young and Guo, 2016; Schallmo et al., 2019). Patients similarly stressed the importance of emotional communication skills in HCW (Reese et al., 2017; Peimani et al., 2020). Looking at this variety of determinants, miscommunication seems likely at some point in the care process.

As safe communication is a professional behavior that is crucial for patient safety and accordingly patient outcomes (Kripalani et al., 2007), one can view communication as health-related behavior. Person-specific characteristics such as self-efficacy or outcome expectancies have been identified as important predictors in behavior change models (Schwarzer et al., 2011; Beeckman et al., 2020). A well-established psychological model explaining health behavior change is the *health action process approach* (HAPA; Schwarzer and Hamilton, 2020). The model suggests that individuals need to form an intention based on outcome expectancies and risk perceptions (motivational phase) before acting accordingly (volitional phase). The pathway of intention on the actual behavior is mediated by action and coping planning. During all phases, self-efficacy is crucial to driving behavior change. The HAPA model has been used to explain a variety of health behaviors and inform interventions (Lippke and Plotnikoff, 2014; Berli et al., 2018; Hamilton et al., 2020), but has not yet been examined in the context of professional communication in healthcare.

In this paper, we aim to examine the HAPA model in the context of professional communication as a health behavior in obstetrics, thus bringing new insights into mechanisms of communication. In a first study, variables from the HAPA model, including outcome expectancies, planning, intention, and action self-efficacy are measured and linked to professional communication. Based on preliminary results from an online pilot survey and the HAPA model, a communication training is developed and tested. According to the proposed structure of social-cognitive variables in the HAPA model, we hypothesize that the HAPA model can explain professional communication and inform a communication intervention. In specific, we hypothesize that:

(1) The HAPA model can explain communication behavior in the healthcare context:

(a) Outcome expectancies and perceived barriers are associated with the intention to communicate safely.
(b) Planning and action self-efficacy mediate the association of the intention to communicate safely with communication behavior.

(2) Perceived patient safety is associated with social-cognitive communication variables, namely outcome expectancies, perceived barriers, intention, self-efficacy, planning, and communication behavior.

(3) The communication intervention increases perceived patient safety, work satisfaction, safe communication behavior, intention, self-efficacy, and planning regarding communication among obstetric HCW, depending on experience and profession.

## Materials and Methods

The research at hand was conducted in two studies. In the first study, social-cognitive variables regarding communication behavior (hypothesis 1a and b), as well as perceived patient safety (hypothesis 2), were measured in a cross-sectional online survey to examine the HAPA model. The second study was built on the first and aimed to replicate the findings regarding the examination of the Health Action Process Approach (hypothesis 1a and b) and perceived patient safety (hypothesis 2). Preliminary results were used to inform a communication intervention for obstetric HCW at two German university hospitals in the second study to create and test a theory- as well as an evidence-based intervention (hypothesis 3). Pre- and post-test data were assessed but no control group was realized due to ethical concerns. Both studies were conducted within the research project “TeamBaby – Safe, digitally supported communication in obstetrics and gynecology” (ClinicalTrials.gov Identifier: NCT03855735) which aims to improve communication among HCW and mothers-to-be and thus enhance patient safety in obstetrics. The project is funded by the German Innovation Fund (Project No. 01VSF18023) of The Federal Joint Committee (G-BA). Details of the research project have been published elsewhere (Lippke et al., 2019).

### Study 1 – Online Survey

#### Participants and Procedure

In the first study, an online survey was conducted via Unipark. The study was frequently (once a month) distributed via press releases, professional associations and social media groups for different occupations within healthcare. At the beginning of 2020, an Email list with quality management representatives from 1,500 hospitals was obtained from a project partner and a request to forward the survey to their employees was sent. The questionnaire consisted of two parts. The first regarded perceived patient safety and participants were asked to fill in the second part regarding communication and associated social-cognitive parts after finishing the first part.

Data was collected from 9th October 2019 to 6th March 2020. Participants were eligible if they indicated that they were more than 18 years of age and worked or were currently being trained in healthcare at least part-time.

#### Measures

A newly developed questionnaire was used to measure communication behavior and social-cognitive determinants. Published and validated scales for social-cognitive determinants in health psychology (Gholami et al., 2016) were adapted to the health behavior of professional communication using the communication competencies by Rider and Keefer (2006). The initial item pool was reviewed by experts in the field and examined in a think-aloud session with three HCW from different fields (appr. 1–10 years of experience). Items with poor parameters [causing a low internal scale consistency (Cronbach’s α < 0.60) or identified as different factors in an exploratory factor analysis] were omitted from further analysis. All statements and factor analyses for the scales can be found in the online Appendix 1. Finally, short scales were included regarding communication behavior (seven items, Cronbach’s α = 0.80), outcome expectancies (three items, Cronbach’s α = 0.76), action self-efficacy (two items, Spearman–Brown coefficient = 0.77) and coping self-efficacy (eight items, Cronbach’s α = 0.82), intention to communicate safely (four items, Cronbach’s α = 0.88), action planning (three items, Cronbach’s α = 0.93) and coping planning (two items, Spearman–Brown coefficient = 0.81) as well as perceived barriers toward safe communication (six items, Cronbach’s α = 0.75). To capture perceptions of patient safety, a trigger for adverse events scales (Keller et al., 2021) was adapted to the perspective of HCW. Participants were asked to rate how often they noticed possible triggers for patient safety incidents on a 30-item scale (Cronbach’s α = 0.96). All variables were measured on six-point Likert scales (1 – “Absolutely not” to 6 – “Absolutely”).

The questionnaires included socio-demographic questions regarding sex, age, profession, and experience. Age was categorized into four groups (“25 years old or younger,” “26–40 years old,” “41–55 years old,” “56 years old or older”). Profession was measured in five groups (“physician,” “midwife,” “nurse,” “midwife or nurse in training,” “other”). Sex and experience were categorized into three groups (“Men,” “Women,” “other” and “less than 1 year,” “1-5 years,” “more than 5 years,” respectively). There was always the option “I’d rather not tell” in case participants were not comfortable answering a question. Participants were not offered any form of compensation for participating in the study.

#### Ethical Approval

Approval for the online survey was given by the Ethics Committee at Jacobs University Bremen (dated September 17, 2019). All participants were informed about the purpose of the study, data security and processing at the beginning of the survey, and asked to indicate their consent explicitly before clicking “continue.”

#### Data Analysis

A path analysis was conducted in R Studio (R version 4.0.3) to estimate and test for associations of the social-cognitive variables (outcome expectancies, perceived barriers, intention, self-efficacy, and planning) with communication behavior (hypotheses 1a and b). Missing data were imputed using Multivariate Imputation by Chained Equations (MICE) which is a principled and flexible method of addressing missing data (Azur et al., 2011). The path analysis was conducted with the “runMI” command from the “semTools” package with 20 imputed datasets. Rubin’s (1987) rules were used to pool parameter estimates, and to calculate degrees of freedom for each parameter test.

To examine the associations of communication behavior and social-cognitive variables with perceived patient safety (hypothesis 2), a multiple linear regression analysis was conducted with the independent variables of communication behavior, outcome expectancies, intention, self-efficacy, planning, and perceived barriers. Sex, profession, and experience were added as dummy-coded variables. Sex and experience were coded as binary variables (“Men” vs. “Women” and “5 years or less” vs. “more than 5 years,” respectively). Profession was coded into four groups to retain higher group sizes and reduce the number of necessary control variables in the analysis. The reference group “nurse” was compared to “midwife,” “physician,” and “other.”

### Study 2 – Communication Trainings at Two Obstetric Hospitals

#### Participants and Procedure

Participants of the second study were all HCW in two German obstetric university hospitals who worked at least part-time in the delivery rooms or postpartum units. Physicians, midwives, and nurses from all hierarchies were required to participate in a training session. Participating in the questionnaires was voluntary. Both hospitals provide the highest level of care with affiliated neonatal intensive care units and have approximately 2,800–3,200 deliveries every year, of which 50% can be classified as medium-to-high risk.

At each hospital, a study nurse and a research associate informed the HCW about the research project including the communication trainings and handed out written information. Informed consent sheets were given to participants together with the baseline questionnaires. Affiliated personnel (e.g., senior consultants and head midwives) helped the recruitment in team meetings and via personal contact. Quality management departments were involved to ensure adequate enrollment and avoid a possible selection bias. HCW were asked to register for a training date and return the baseline questionnaire via closed boxes in staff rooms in the delivery and postpartum units. Participants were occasionally reminded to fill in the questionnaire via email, personal contact, or short notes. After all training sessions were completed, participants were asked to fill in the post-intervention questionnaire which was matched to the baseline data via a unique participant code.

Data was collected from 2nd January to 16th October 2020. However, data collection and training sessions had to be paused from 16th March to 17th June due to COVID-19 regulations issued by the local health authorities. An intervention group only study design was implemented because spill-over effects were likely to occur. The main aim of the research project was to enhance patient safety. Since an important outcome was supposed to be a reduction in pAE extracted from routine hospital data, the training was mandatory for all HCW working at least part-time in the obstetric units. Temporary team members such as midwives-in-training and those working in the obstetric units with fewer hours were invited to take part. Study codes were checked to make sure that none of the HCW had taken part in the online survey.

#### The TeamBaby Safe Communication Intervention

The TeamBaby safe communication trainings were developed by a company for trainings targeting patient safety in obstetrics in close cooperation with the project team consisting of health psychologists, public health experts, sociologists, and obstetric healthcare professionals. To make the intervention more feasible within the demanding setting of obstetric care at the university hospitals, and at the same time ensure effective transfer, a 4-h in-person training session was agreed upon and followed by: individual behavior planning, pocket cards as reminders as well as biweekly microteaching units via an online tool. In total, 13 trainings were conducted as in-person trainings at the respective hospitals (seven in hospital 1 and six in hospital 2).

The training was theoretically based on the preliminary results from the online survey and the HAPA model. Social-cognitive constructs that were found to be associated according to model suggestions (e.g., the intention to communicate safely) were included in the training. For planning, it was decided to include a more explicit intervention, namely the behavioral planning intervention sheet at the end of the training since communication planning seemed to be less intuitive. The preliminary results were derived from a general HCW sample in Germany and not an obstetric one. To still address the peculiarities of obstetrics, trainers with a background in obstetrics (two former midwives and an anesthesiologist with long experience in obstetric units) developed the training session. One of the midwives and the anesthesiologist served as the trainers. Finally, the training consisted of a short introduction round to recognize different mental models regarding a “good” birth, a film to strengthen the awareness toward the role of communication as well as different exercises to teach closed-loop communication, speaking-up, structured handovers, perspective taking toward other team members and the mothers-to-be, as well as different communication competencies such as the adaption to different addressees and situations.

An overview of all training contents, exercises and goals, related behavior change techniques as well as the behavioral planning intervention and microteachings is provided in the online Appendices 2–4.

#### Measures

Based on preliminary results from the online survey, the questionnaire was shortened and adapted for application at the university hospitals for baseline and post-intervention measurements. Items with poor parameters in the online survey were omitted from the questionnaire and items with unsatisfactory parameters in the hospital study were excluded prior to the analyses. HCW and their superiors voiced concerns over the time that the questionnaires would require so that it was decided to substantially shorten the questionnaires and use single-item scales where possible. All statements can be found in the online Appendix. The final measures for the training evaluation at the hospitals were composed of short scales regarding communication behavior (seven items, Cronbach’s α at baseline = 0.85/post-intervention 0.89), outcome expectancies (three items, Cronbach’s α = 0.73), coping self-efficacy (four items, Cronbach’s α = 0.78/0.81), and perceived barriers (three items, Cronbach’s α = 0.66). Action self-efficacy, intention to communicate safely, action planning, and coping planning were measured as single-item scales at both time points. The trigger for adverse events scale was shortened to seven items so that the multi-faceted construct could still be measured in more detail (Cronbach’s α = 0.79/0.87). Work satisfaction was measured with a short version of the Brayfield and Rothe (1951) scale, which has been shown to be reliable and valid (Judge et al., 2002). One item was deleted after the examination so that three items remained (Cronbach’s α = 0.92/0.92). Answer formats and questions regarding demographic data were the same as in the online survey. Categorical data was used to ensure anonymity at the hospitals.

A feasibility questionnaire was used to capture participants’ initial reactions and acceptance of the training. The questionnaire asked about HCWs’ experience with the training concerning overall conditions (five items, Cronbach’s α = 0.75), the trainers (one item), training contents (nine items, Cronbach’s α = 0.86), benefits of the training (eight items, Cronbach’s α = 0.90), and overall acceptability (three items, Cronbach’s α = 0.86). All answers were given on a four-point smiley scale (1 – two negative smileys to 4 – two positive smileys). An open question for further comments was added.

#### Ethical Approval

Ethical approval for the data collection and training sessions at the obstetric hospitals was granted as part of the TeamBaby ethical approval from the University Hospital of Ulm Human Research Ethics Committee (Number 114/19) and the University Hospital of Frankfurt Medical Research Ethics Committee (Number 19-292). All study participants provided written informed consent to participate in the study. Providing data via questionnaires was voluntary, but senior consultants and head midwives at both hospitals decided that the trainings were mandatory. Participants were not offered any form of compensation for participating in the study.

#### Data Analysis

Both the path and regression analyses as described in Study 1 were replicated with baseline data (hypotheses 1a/b, 2). In the regression analysis, hospital was added as an additional control variable.

Means and standard deviations concerning feasibility and acceptance are reported descriptively. For the analysis of open answers, a thematic analysis was conducted (Braun and Clarke, 2006). It was chosen to look for themes in the short sentences or comments provided by the HCW. After reading all open answers, the main author categorized them according to their content into three main contents identified in the first reading: “positive comments,” “criticism,” and “further suggestions to improve the trainings.” An open answer could be grouped in as many as all three categories if it contained more than one theme. It was decided after the first reading that the category of positive comments should include appreciative statements, the indication of positive feelings or notes of thanks. The category of criticism included statements of disapproval or disappointment as well as comments of missing topics in the training if no clear suggestion was given. Specific suggestions regarding the mode of delivery, content of the training and its timing were included in the further suggestions to improve the training. Percentages were calculated and reported based on the number of HCW who provided an answer to the open question.

To examine hypothesis 3, time and group differences regarding safe communication behavior, perceived patient safety, work satisfaction and social-cognitive variables were tested in a repeated-measures MANOVA with the independent variables hospital, profession, and experience. The factor “time” refers to the differences between the baseline measure (t1) and the post-intervention questionnaire (t2) aggregated across groups (e.g., profession or hospital). Group differences are aggregated over the two time points while the interaction effect (time*group) shows the group differences in the changes over time. Participant groups were partly summarized due to small sample sizes. Profession was coded into three groups (“physician,” “midwife” and “other”), and experience was categorized dichotomously (“5 years or less” and “more than 5 years”). Sex was not added to the model since most HCW in the obstetric units were female (91%). Age was omitted due to a high correlation with experience (Spearman’s ρ = 0.64, *p* < 0.001) to avoid multicollinearity. Interaction effects between time and respectively hospital, profession, and experience were tested, but higher-level interaction effects were omitted from the model since group combinations were too small. A *post hoc* power analysis for the MANOVA was calculated with G*Power for multivariate global effects for a predictor with three groups and an effect size of *f*^2^ = 0.15 as well as for univariate interaction effects for a predictor with three groups and an effect size of *f* = 0.4.

Participants from the second study were compared to those from the first study with χ^2^-tests. Participants who dropped out were compared to those who completed the post-intervention questionnaire using *t*-tests for independent samples and χ^2^-tests. All data analysis for the second study was carried out using R Studio (R version 4.0.3) and IBM SPSS Statistics Version 27.

## Results

### Study 1

#### Participants

Two thousand one hundred and two persons clicked on the link for the online questionnaire. *N* = 505 (26.6%) advanced beyond the introduction page and indicated their informed consent. Three were test subjects who filled in the questionnaire in a think-aloud session and were thus excluded. Of the *N* = 502 participants who gave their consent, *N* = 173 (34.5%) finished the first part regarding perceived patient safety and *N* = 130 (25.9%) finished both parts. One participant was excluded who rated every single item with the highest possible value. Thus, data from *N* = 129 HCW were used in the analysis. Demographic data is provided in Table 1.

**TABLE 1:** Demographic data.

		Study 1 – Online survey (*N* = 129)	Study 2 – Communication trainings at obstetric hospitals (*N* = 137)	Test statistics		
				χ ^2^	*df*	*p*
Sex	Male	34(26%)	11(8%)	20.70	3	<.001
	Female	91(71%)	124(91%)			
	Diverse	1(1%)	0(0%)			
	Missing	3(2%)	2(2%)			
Age	Younger than or 25 years old	8(6%)	29(21%)	43.69	4	<.001
	26–40 years old	46(36%)	75(54%)			
	41–55 years old	46(36%)	21(15%)			
	56 years old or older	26(20%)	6(4%)			
	Missing	3(2%)	6(4%)			
Profession	Physician	18(14%)	45(33%)	49.26	6	<.001
	Midwife	14(11%)	45(33%)			
	Nurse	58(45%)	23(17%)			
	Nurse/midwife in training	0(0%)	11(8%)			
	Other	24(19%)	11(8%)			
	Missing	13(10%)	2(2%)			
Experience	<1 year	2(2%)	21(15%)	45.76	3	<.001
	1–5 years	23(18%)	56(41%)			
	>5 years	103(80%)	54(39%)			
	Missing	1(1%)	6(4%)			

*The “other” category for profession includes psychologists, physiotherapists, quality/ward managers and social workers.*

#### Examination of the Health Action Process Approach (Hypotheses 1a and b)

**FIGURE 1:**
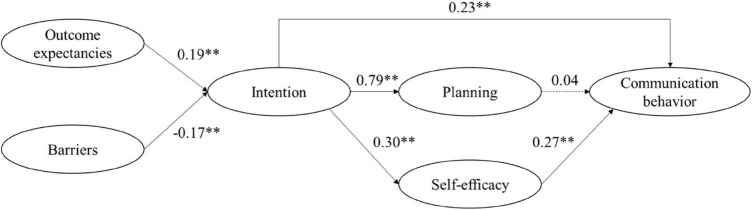
Path model derived from the HAPA model to establish associations between social-cognitive communication variables in an online HCW sample (Study 1). Coefficients are reported as unstandardized. Fit indices: χ^2^(*df* = 7) = 8.86, *p* = 0.263; *CFI* = 0.98, *RMSEA* = 0.05, *CRMR* = 0.06; ^**^; *p* < 0.01.

The results indicated a good model fit [χ^2^(*df* = 7) = 8.86, *p* = 0.263; *CFI* = 0.98, *RMSEA* = 0.05, *CRMR* = 0.06] of the path model (Figure 1). Missing data were less than 1.6% for all variables (0.87% on average). Outcome expectancies (β = 0.30, *p* < 0.001) and perceived barriers to safe communication (β = –0.26, *p* = 0.002) were associated with the intention to communicate safely. Intention had a positive association with behavior planning (β = 0.45, *p* < 0.001) and self-efficacy (β = 0.24, *p* = 0.005). Better communication was associated with intention (β = 0.27, *p* = 0.002) and self-efficacy (β = 0.38, *p* < 0.001), but not planning (β = 0.08, *p* = 0.335). There was no significant indirect effect from the intention to communicate safely to the communication behavior via planning (α*β = 0.04, *p* = 0.340), but self-efficacy mediated the association (α*β = 0.09, *p* = 0.014).

#### Perceived Patient Safety (Hypothesis 2)

The multiple regression on perceived patient safety displayed that self-efficacy was related to less perceived triggers and thus higher patient safety (β = –0.26, *p* = 0.01), whereas planning (β = 0.21, *p* = 0.045) and barriers of effective communication (β = 0.24, *p* = 0.013) were associated with more triggers and thus lower patient safety. All results are displayed in Table 2.

**TABLE 2:** Regression analysis of social-cognitive communication variables on perceived patient safety in the online HCW sample.

	Unstandardized coefficients			Standardized coefficients	
	*B*	*SE*	β	*t*	*p*
Communication behavior	–0.21	0.17	–0.14	–1.26	0.210
Outcome expectancies	0.05	0.09	0.05	0.56	0.580
Intention	–0.04	0.16	–0.03	–0.26	0.792
Self-efficacy	–0.27	0.10	–0.26	–2.65	0.010
Planning	0.16	0.08	0.21	2.03	0.045
Barriers	0.22	0.09	0.24	2.53	0.013
Sex	0.05	0.16	0.03	0.32	0.750
Experience	–0.12	0.21	–0.06	–0.55	0.582
Profession 1^1^	0.12	0.24	0.05	0.49	0.622
Profession 2^1^	0.01	0.21	0.01	0.07	0.949
Profession 3^1^	–0.34	0.18	–0.17	–1.89	0.061

*Perceived patient safety was assessed as triggers for adverse events. Negative regression coefficients therefore mean less triggers and thus higher perceived patient safety.*

*^1^Profession 1: comparison between nurses and midwives, Profession 2: comparison between nurses and physicians, Profession 3: comparison between nurses and “others.”*

### Study 2

#### Participants

Of the *N* = 141 HCW who completed the intervention, *N* = 137 HCW provided information on the baseline measures (t1), of which *N* = 73 (53.3%) came from the first and *N* = 64 (46.7%) came from the second hospital. *N* = 87 (63.5%) completed the post-intervention questionnaire (t2) from July to October of which 69 could be matched to baseline measures based on study codes (50.4%). Compared to the sample from the first study, it became apparent that participants from the second study were younger [χ^2^(df = 4) = 43.69, *p* < 0.001], less experienced [χ^2^(df = 3) = 45.76, *p* < 0.001], more predominantly female [χ^2^(df = 3) = 20.70, *p* < 0.001] and that there were more physicians and midwives than nurses [χ^2^(df = 6) = 49.26, *p* < 0.001].

Demographic data and the sample comparison are provided in Table 1.

#### Replication of the Health Action Process Approach (Hypotheses 1a and b)

**FIGURE 2:**
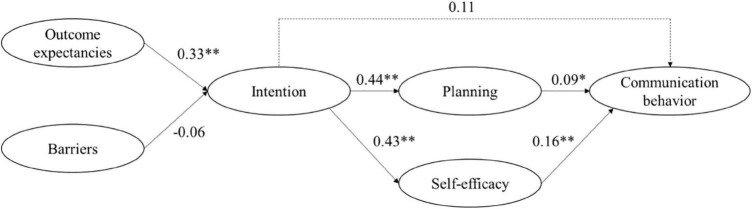
Path model derived from the HAPA model to establish associations between social-cognitive communication variables in an obstetric HCW sample at two university hospitals (Study 2). Coefficients are reported as unstandardized. Fit indices: χ^2^(*df* = 7) = 19.00, *p* = 0.008; *CFI* = 0.77, *RMSEA* = 0.11, *CRMR* = 0.10; *p < 0.05; ^**^p < 0.01.

Missing data was less than 14.9% for all variables and 10.87% on average in the baseline measures. Results indicated a problematic model fit [χ^2^(*df* = 7) = 19.00, *p* = 0.008; *CFI* = 0.77, *RMSEA* = 0.11, *CRMR* = 0.10] for the replicated path model (Figure 2). Outcome expectancies (β = 0.24, *p* = 0.004) but not barriers (β = –0.06, *p* = 0.362) were associated with the intention to communicate safely. Intention revealed a positive association with behavior planning (β = 0.24, *p* = 0.006) and action self-efficacy (β = 0.32, *p* < 0.001). Better communication was associated with behavior planning (β = 0.18, *p* = 0.035) and action self-efficacy (β = 0.24, *p* = 0.006) but not intention (β = 0.13, *p* = 0.153). There was no significant indirect effect from intention to the actual behavior via planning (α*β = 0.04, *p* = 0.131) but action self-efficacy mediated the association (α*β = 0.08, *p* = 0.028).

#### Replication of the Perceived Patient Safety Regression Analysis (Hypothesis 2)

The replicated multiple regression on perceived patient safety displayed that neither action self-efficacy (β = 0.02, *p* = 0.845) nor planning (β = 0.00, *p* = 0.996) were related to less perceived triggers and thus higher patient safety, whereas barriers of effective communication (β = 0.22, *p* = 0.013) were associated with more triggers and thus lower patient safety. Communication behavior was related to less perceived triggers (β = –0.41, *p* < 0.001). There were significant differences in perceived patient safety between hospitals (β = –0.26, *p* = 0.014). All results are displayed in Table 3.

**TABLE 3:** Regression analysis of social-cognitive communication variables on perceived patient safety in the obstetric HCW sample from the university hospitals.

	Unstandardized coefficients			Standardized coefficients	
	*B*	*SE*	β	*t*	*p*
Communication behavior	–0.43	0.11	–0.41	–3.90	<0.001
Outcome expectancies	–0.13	0.11	–0.10	–1.12	0.264
Intention	–0.07	0.09	–0.07	–0.79	0.429
Self-efficacy	0.01	0.07	0.02	0.20	0.845
Planning	0.00	0.04	0.00	–0.01	0.996
Barriers	0.16	0.07	0.22	2.53	0.013
Hospital	–0.36	0.15	–0.26	–2.50	0.014
Sex	0.15	0.23	0.06	0.65	0.515
Experience	–0.11	0.09	–0.11	–1.32	0.190
Profession 1^1^	0.05	0.14	0.03	0.36	0.719
Profession 2^1^	–0.18	0.16	–0.10	–1.09	0.278
Profession 3^1^	–0.12	0.20	–0.06	–0.60	0.550

*Perceived patient safety was assessed as triggers for adverse events. Negative regression coefficients therefore mean less triggers and thus higher perceived patient safety.*

*^1^Profession 1: comparison between nurses and midwives, Profession 2: comparison between nurses and physicians, Profession 3: comparison between nurses and “others.”*

#### Feasibility and Acceptability of the Training

In total, 128 HCW answered the feasibility questionnaire. Overall, the intervention was rated with *M* = 3.3 out of 4 (*SD* = 0.54). The trainers (*M* = 3.52, *SD* = 0.63) and overall conditions (*M* = 3.51, *SD* = 0.44) were rated the highest, but training contents (*M* = 3.12, *SD* = 0.47) and benefits (*M* = 3.09, *SD* = 0.49) were still rated positively. Eight HCW (6.3%) gave negative ratings (*M* < / = 2.5) for the training, 1 HCW (0.8%) rated the overall conditions negatively, 5 (3.9%) disliked the moderators, 15 (11.7%) were not satisfied with the contents and 17 (13.3%) doubted that the trainings had benefits for their job.

Twenty HCW answered the open question. Eight (40%) wished to thank the trainers or left a positive comment (e.g., “Everything was wonderful”) whereas 4 (20%) criticized the training as too short or missing important topics (e.g., “The most fundamental part of communication problems was not incorporated. Language barriers are from the experience the biggest problem”). Twelve HCW (60%) had suggestions for future trainings including more frequent training sessions, simulation training, and more content about emergency situations and techniques to communicate with “difficult” patients (e.g., “I wish for more training on emergency situations, trainings appr. 1 time in 6 months”).

#### Intervention Effects (Hypothesis 3)

Due to the high drop-out and not matchable questionnaires, missing data were 45–47% for post-intervention scales and could not be imputed. The power analysis indicated that the power for the MANOVA was sufficient to detect medium sized effects (multivariate global effects with a power of 76% and univariate interaction effects with a power of 82%). The repeated-measures MANOVA is reported in Table 4. It showed multivariate significant between-subjects effects for hospital [*F*(*df* = 8) = 4.28, *p* = 0.001, η_*p*_^2^ = 0.46] and experience [*F*(*df* = 8) = 4.79, *p* < 0.001, η_*p*_^2^ = 0.48] as well as a significant within-subject effect for time [*F*(*df* = 8) = 2.92, *p* = 0.011, η_*p*_^2^ = 0.36]. For univariate between-subjects effects (Table 5A), the hospital had a significant effect on communication behavior [*F*(*df* = 1) = 18.36, *p* < 0.001, η_*p*_^2^ = 0.28], the intention to communicate safely [*F*(*df* = 1) = 4.12, *p* = 0.048, η_*p*_^2^ = 0.08] and action planning [*F*(*df* = 1) = 5.20, *p* = 0.027, η_*p*_^2^ = 0.10] aggregated over time points. Less experience was associated with higher perceived patient safety risks [*F*(*df* = 1) = 7.53, *p* = 0.009, η_*p*_^2^ = 0.14] and lower work satisfaction [*F*(*df* = 1) = 4.88, *p* = 0.032, η_*p*_^2^ = 0.09], whereas profession did not have any significant effects when averaged over all time points.

**TABLE 4:** Multivariate MANOVA results.

	Between subjects					
	Wilks–Lambda	*F*	Hypothesis *df*	Error *df*	*p*	η _*p*_^2^
Hospital	0.55	4.28	8	41	0.001	0.46
Profession	0.66	1.18	16	82	0.303	0.19
Experience	0.52	4.79	8	41	< 0.001	0.48

	**Within subjects**					
	
	**Wilks-Lambda**	***F***	**Hypothesis *df***	**Error *df***	***p***	**η _*p*_^2^**

Time	0.637	2.92	8	41	0.011	0.36
Hospital*Time	0.760	1.62	8	41	0.150	0.24
Profession*Time	0.702	0.99	16	82	0.472	0.16
Experience*Time	0.749	1.72	8	41	0.124	0.25

*Multivariate tests for perceived patient safety risks, work satisfaction, communication behavior, intention, action and coping self-efficacy, action planning and coping planning.*

**TABLE 5A:** Univariate between-subjects MANOVA results.

		Between subjects				
		Type III SS	*df*	*F*	*p*	η _*p*_^2^
Hospital	Perceived patient safety risks	0.32	1	0.43	0.516	0.01
	Work satisfaction	0.86	1	0.46	0.500	0.01
	Communication behavior	9.38	1	18.36	<0.001	0.28
	Intention	3.03	1	4.12	0.048	0.08
	Action self-efficacy	0.06	1	0.07	0.792	< 0.01
	Coping self-efficacy	0.83	1	0.90	0.348	0.08
	Action planning	16.86	1	5.20	0.027	0.10
	Coping planning	0.94	1	0.87	0.357	0.02
Profession	Perceived patient safety risks	0.92	2	0.63	0.539	0.03
	Work satisfaction	3.69	2	1.00	0.377	0.04
	Communication behavior	1.33	2	1.30	0.281	0.05
	Intention	1.56	2	1.08	0.348	0.04
	Action self-efficacy	0.23	2	0.13	0.877	0.02
	Coping self-efficacy	3.56	2	1.93	0.157	0.07
	Action planning	11.66	2	1.80	0.176	0.07
	Coping planning	0.22	2	0.10	0.906	< 0.01
Experience	Perceived patient safety risks	5.55	1	7.53	0.009	0.14
	Work satisfaction	9.05	1	4.88	0.032	0.09
	Communication behavior	0.08	1	0.15	0.704	< 0.01
	Intention	0.09	1	0.13	0.725	< 0.01
	Action self-efficacy	1.33	1	1.49	0.229	0.03
	Coping self-efficacy	0.40	1	0.44	0.513	0.01
	Action planning	1.78	1	0.55	0.463	0.01
	Coping planning	1.87	1	1.73	0.195	0.04

*Between-subjects tests for perceived patient safety risks, work satisfaction, communication behavior, intention, self-efficacy, coping self-efficacy, action planning, and coping planning.*

Concerning univariate within-subject effects (Table 5B), time had an influence on perceived patient safety risks [*F*(*df* = 1) = 10.67, *p* = 0.002, η_*p*_^2^ = 0.18] and coping self-efficacy [*F*(*df* = 1) = 4.39, *p* = 0.041, η_*p*_^2^ = 0.08]. The interaction effect of hospital and time was significant for communication behavior [*F*(*df* = 1) = 7.62, *p* = 0.008, η_*p*_^2^ = 0.14], and the interaction between experience and time was significant in coping planning [*F*(*df* = 1) = 7.05, *p* = 0.011, η_*p*_^2^ = 0.13]. While HCW with less than 5 years of experience showed an improvement in coping planning in the post-intervention time point compared to baseline (*M*_pre–intervention_ = 4.16, *SD* = 0.95; *M*_post–intervention_ = 4.58, *SD* = 0.89), HCW with more than 5 years of experience had lower scores than before the intervention (*M*_pre–intervention_ = 4.83, *SD* = 0.83; *M*_post–intervention_ = 4.48, *SD* = 1.18).

**TABLE 5B:** Univariate within-subjects MANOVA results.

		Within subjects				
		Type III SS	*df*	*F*	*p*	η _*p*_^2^
Time	Perceived patient safety risks	3.13	1	10.67	0.002	0.18
	Work satisfaction	0.14	1	0.43	0.515	0.01
	Communication behavior	0.06	1	0.27	0.606	0.01
	Intention	0.13	1	0.28	0.601	0.01
	Action self-efficacy	1.33	1	1.46	0.233	0.03
	Coping self-efficacy	1.95	1	4.39	0.041	0.08
	Action planning	0.27	1	0.40	0.529	0.01
	Coping planning	0.01	1	0.01	0.910	< 0.01
Time*Hospital	Perceived patient safety risks	0.25	1	0.86	0.358	0.02
	Work satisfaction	0.00	1	0.01	0.938	< 0.01
	Communication behavior	1.62	1	7.62	0.008	0.14
	Intention	0.42	1	0.91	0.345	0.02
	Action self-efficacy	0.15	1	0.16	0.690	< 0.01
	Coping self-efficacy	0.18	1	0.41	0.528	0.01
	Action planning	0.01	1	0.02	0.891	< 0.01
	Coping planning	0.42	1	0.90	0.347	0.02
Time*Profession	Perceived patient safety risks	1.20	2	2.04	0.141	0.08
	Work satisfaction	0.74	2	1.14	0.328	0.05
	Communication behavior	0.61	2	1.44	0.247	0.06
	Intention	0.64	2	0.70	0.502	0.03
	Action self-efficacy	1.03	2	0.57	0.570	0.02
	Coping self-efficacy	0.82	2	0.92	0.407	0.04
	Action planning	1.57	2	1.16	0.323	0.05
	Coping planning	1.33	2	1.42	0.252	0.06
Time*Experience	Perceived patient safety risks	0.14	1	0.46	0.501	0.01
	Work satisfaction	0.06	1	0.18	0.677	< 0.01
	Communication behavior	0.02	1	0.10	0.750	< 0.01
	Intention	0.59	1	1.27	0.265	0.03
	Action self-efficacy	0.04	1	0.04	0.838	< 0.01
	Coping self-efficacy	0.01	1	0.02	0.904	< 0.01
	Action planning	1.39	1	2.06	0.158	0.04
	Coping planning	3.31	1	7.05	0.011	0.13

*Within-subjects tests for perceived patient safety risks, work satisfaction, communication behavior, intention, self-efficacy, coping self-efficacy, action planning, and coping planning.*

#### Drop-Out Analysis

Group comparisons showed that participants who completed the post-intervention questionnaire did not differ from those who dropped out concerning the hospital, age, sex, and experiences or the target variables [all *p*-values > 0.05 for perceived patient safety risks, work satisfaction, communication behavior, intention to communicate safely, (coping) self-efficacy, action/coping planning]. However, participants who completed the questionnaire were less likely to be nurses than participants who dropped out [11% of those who completed the post-intervention measure were nurses vs. 26% of those who did not; χ^2^(*df* = 4) = 11.67, *p* = 0.020].

## Discussion

Answering hypotheses 1a and b, both studies showed that most relations proposed in the health action process approach could be found in the context of communication in healthcare, hence indicating that the HAPA can be used to explain safe communication behavior. Regarding the first part of the hypothesis, the intention to communicate safely was significantly related to outcome expectancies and perceived barriers. Concerning the second part, the association between intention and communication behavior was mediated by self-efficacy. However, the role of planning was less clear since it was associated with communication behavior only in the second study.

Regarding hypothesis 2, self-efficacy was related to fewer triggers of adverse events while barriers were associated with more triggers in the first study. Surprisingly, planning of effective communication was related to more triggers indicating a lower perceived patient safety. Communication behavior was only directly related to perceived patient safety in the second study so that hypothesis 2 cannot be fully supported.

Concerning hypothesis 3 regarding intervention effects, improvements in perceived patient safety, as well as coping self-efficacy, could be found. However, the target behavior of safe communication did not change. In subgroup analyses, it was found that communication behavior differed between the respective hospitals (communication behavior developed differently over time in the different hospitals). Higher work experience was associated with fewer perceived risks and higher satisfaction. HCW with only a short work experience showed an improvement in coping planning, whereas HCW with more experience decreased in their coping planning.

Communication trainings can be effective to improve patient safety (Lippke et al., 2021). However, the consideration of patient safety and communication in the German health care system is lacking behind many other countries (Schrappe et al., 2020). Nevertheless, HCW adapt their safety strategies to imperfect working conditions including increasing patient-staff ratios, economic constraints, and frequent staff turnover (Douglas et al., 2017). This background must be considered when interpreting the current results.

On a very positive side, both studies suggest that HCW are aware of the impact of safe communication on patient safety. Perceived triggers were related to higher planning of safe communication and communication behavior, indicating that HCW perceived the need to act upon perceived patient safety risks. The results concerning planning might appear unexpected at first glance but can be explained by HCWs’ awareness. HCW who perceived more safety risks also felt a greater need to plan their communication accordingly.

While Study 1 addressed HCW from all fields of patient care and medicine via an online survey, participants of Study 2 were all HCW in obstetrics who participated in a communication training session of 4 hours. No difference in the target behavior of safe communication could be revealed; however, perceived patient safety improved. A simple ceiling effect might explain why communication behavior did not improve. Since communication was reported to be very high already before the training session, HCW might not have been able to indicate positive changes in the post-intervention questionnaire. It remains questionable, however, whether the ratings of communication behavior were high due to HCWs’ prior exposure to communication interventions in the university hospitals or whether they overestimated their communication skills.

One interesting finding is the significant difference between the two clinics regarding perceived patient safety in the regression analysis for Study 2. Similar differences were found in the MANOVA regarding communication and its change over time as well as the intention to communicate safely and action planning. A cause cannot be determined as a variety of organizational, team climate, or work policies may account for the differences. Indeed, they indicate the need for further research considering different sites and safety cultures. For example, it is possible that communication behavior was not necessarily better at one site but that HCW at the other hospital were more confident in voicing concerns (Morrow et al., 2016). Another surprising finding is that we failed to identify differences between professional groups regarding perceived patient safety, communication behavior and social-cognitive variables, which is not consistent with the literature (Foronda et al., 2016). The only significant difference between professional groups was that nurses were less likely to fill in the post-intervention questionnaire when compared to other groups. This might be because the training was focused on the birth situation, in which midwives and physicians play a bigger role. Hence, nurses might have perceived that the training was less suitable for their professional group and thus had lower motivation to finish the post-intervention questionnaire.

Subgroup analyses of participants with shorter work experience showed improvements in coping planning from baseline to post-intervention, while those with more than 5 years of work experience did not. As presented in similar research, encouragement in speaking-up as was trained, is highly appreciated by HWC in younger professional age (Schmiedhofer et al., 2021). In contrast, HCW with longer work experience perceived fewer patient safety risks and higher work satisfaction after the intervention, but also reported lower coping planning. Since they have more routine behavior, they are less likely to be affected by the intervention and change their communication behavior (Braithwaite et al., 2015). The decrease in coping planning indicates an increased awareness of their own behavior. Participants may have established a new or deeper understanding of communication in the context of patient safety after being trained.

The HAPA model may present some explanations for initially surprising results, as far as planning and self-efficacy are concerned. The HAPA model was developed to support people in changing their personal, clearly defined, and measurable health behavior, e.g., smoking cessation or change in nutrition intake (Lippke and Plotnikoff, 2014; Berli et al., 2018). In contrast, communication is not only a complex interpersonal behavior, but success is also more difficult to quantify. Neither in the online survey nor the communication intervention at the obstetric hospitals, was the HAPA model described in detail. Therefore, a more detailed training unit regarding behavior planning and maintenance might have been necessary to increase effects. This assumption is confirmed by some HCW who asked for a (repeated and regular) follow-up of the training session in the open feasibility question.

When interpreting the results of this study, several limitations must be borne in mind. We were not able to realize a randomized-controlled trial with a control group due to possible spill-over effects and the aim to provide the highest possible level of care to all expectant mothers. Therefore, we cannot rule out alternative explanations. Since the intervention study was conducted during the COVID-19 pandemic in Germany, its influences must be considered when interpreting the results. Previous research has shown that deficits in self-efficacy may be related to fears associated with the COVID-19 pandemic, reduced social support, and an increase in perceived stress and anxiety among HCW (Badrfam et al., 2020). It is possible that the communication intervention and consequent staff efforts have buffered the negative effects of the COVID-19 pandemic. On the other hand, this brief intervention may not be sufficient to sustain improved communication in the longer term. Due to the restrictions by local health authorities, there was a long period of time between questionnaires and the training. To capture all improvements in communication, more frequent time points of measurement including a follow-up would have been needed but could not be realized.

Additionally, communication was assessed in newly developed self-reports which revealed surprisingly high levels of pre-intervention communication. Both university hospitals have regular trainings and report a high awareness for communication, which could have caused a good level of communication prior to our study. Nevertheless, it is also plausible that HCW did not feel safe enough to indicate low levels of their communication since their superiors were part of the project team. This would be more indicative of social desirability or a lack of a safety culture. It remains questionable whether self-reported communication behavior was a good indicator. Including interviews or observations could have provided additional information on how the training affected communication competencies. Hence, future research with more objective and sensitive-to-change measures is needed.

While participants in Study 1 were recruited from various medical disciplines, the intervention in Study 2 was explicitly designed for the department of obstetrics. Participants from different disciplines may have different communication characteristics. Hence, the intervention in Study 2 might have been of a higher quality if it was based on preliminary results from the same field and from a more similar sample. In addition, it seems likely that the sample from the first study was selective when looking at the response rates. It is possible that only HCW who had a high interest in and were particularly aware of the crucial role of communication finished the questionnaire. Nevertheless, communication is an integral part of healthcare in every field (Howick et al., 2018), so that we assumed that the results from the online survey could inform the communication intervention in Study 2.

Other methodological limitations to the results include the use of single-item scales to ensure feasibility under time pressure. However, single-item scales need to be treated with caution because they might not be appropriate for heterogeneous constructs and have a lower reliability. Concerning the replication of findings from the path analysis in the second study, it must be noticed that the model fit was problematic. Furthermore, the high drop-out rate and therefore small sample size in Study 2 poses a power issue for the intended analyses. For the MANOVA, only medium effect sizes could be detected with sufficient power, so that future research with higher sample sizes is needed. Control analyses showed that the drop-outs and the study sample did not differ systematically in the communication variables or socio-demographic characteristics, indicating that the drop-out seems to be unsystematic. Likely reasons for the high drop-out include the COVID-19 pandemic that increased the time between measures. Hence, HCW might not have seen the post-intervention measures as important since the intervention was not as salient. Both hospitals were teaching hospitals with a rather high staff turnover so that some HCW might have been lost for post-intervention questionnaires. Another possible explanation is that HCW needed too much time to fill in the questionnaires and thus decided against it after they received the training intervention which might have acted as an incentive before.

In summary, the research at hand has practical implications regarding the use of the HAPA model to inform communication interventions regarding patient safety. In future research on communication interventions and patient safety in obstetrics, high-quality study designs should be used to evaluate communication trainings. A randomized-controlled trial (RCT) with follow-up measures, reliable change measures and a control group should be applied. Furthermore, future research should include more hospitals and aim to reduce dropout rates to increase the number of participants. Although the intervention was conceptualized as a short training to make it more feasible in the healthcare setting, it might not have been comprehensive enough to cause a long-term improvement in communication behavior. Therefore, future trainings should carefully plan their interventions in close cooperation with all stakeholders at their target hospitals to target pAE and negative consequences for expectant mothers, children and HCW. Different situations could be trained and simulated to achieve an even better understanding on the part of the participants. Online trainings might be promising since they provide the educational contents interactively and flexibly (Blake et al., 2020). This is especially true for the current COVID-19 pandemic during which HCW all over the world have to deal with new challenges and requirements (Lotfinejad et al., 2020; Yang et al., 2020). Buffering negative effects from the pandemic will be a challenge over the coming years. Short and feasible training programs might be a way to help sustain an effective communication.

## Data Availability Statement

The data for this study are not publicly available due to data protection guidelines. The data are available on request from the corresponding author.

## Ethics Statement

The studies involving human participants were reviewed and approved by approval for the online survey was given by the Ethics Committee at Jacobs University Bremen (dated September 17, 2019). Ethical approval for the data collection and training sessions at the obstetric hospitals was granted as part of TeamBaby ethical approval from the University Hospital of Ulm Human Research Ethics Committee (Number 114/19) and the University Hospital of Frankfurt Medical Research Ethics Committee (Number 19-292). The participants provided their written informed consent to participate in this study.

## Author Contributions

SL, CD, LK, and FK contributed to conception and design of the study and supervised the data collection and interpreted the analyses. CD performed the statistical analysis and wrote the first draft of the manuscript. CD, LK, FK, SL, and MS wrote sections of the manuscript. All authors contributed to manuscript revision, read, and approved the submitted version.

## Conflict of Interest

The authors declare that the research was conducted in the absence of any commercial or financial relationships that could be construed as a potential conflict of interest.

## Publisher’s Note

All claims expressed in this article are solely those of the authors and do not necessarily represent those of their affiliated organizations, or those of the publisher, the editors and the reviewers. Any product that may be evaluated in this article, or claim that may be made by its manufacturer, is not guaranteed or endorsed by the publisher.

## Abbreviations

COVID-19: coronavirus disease 2019
HAPA: health action process approach
HCW: healthcare workers
MANOVA: multivariate analysis of variance
MICE: multivariate imputation by chained equations
pAE: preventable adverse events
RCT: randomized-controlled trial.
