# Supplementary material for: Psychological Intervention to Improve Communication and Patient Safety in Obstetrics: Examination of the Health Action Process Approach
Source: Front Psychol. 2022 Feb 18;13:771626. doi: 10.3389/fpsyg.2022.771626 (PMC8894763; doi:10.3389/fpsyg.2022.771626)
Supplement: Supplementary file 1 [file Table_1.DOCX]

**Appendix**

**Appendix 1. Items and scales.**

Cronbach’s α for the online sample/ hospital sample at T1/ hospital sample at T2

Poor item parameters: Item causes a low internal scale consistency (Cronbach’s α <.60) or was identified as different factor in an exploratory factor analysis

Exploratory factor analyses: Principal Component Analysis with Varimax rotation

Items were translated with DeepL

| **Scale** | **Statement** | **Omitted from analyses in online sample (study 1)** | **Changed/ retained for the obstetric university sample (study 2)** | **Cronbach’s α/ or Spearman-Brown Coefficient for study 1/ study 2 T1/ study 2 T2** | **Exploratory factor analyses with retained items** |
| --- | --- | --- | --- | --- | --- |
| **Communication behavior** |  | All items were used in the analyses | Used as in online sample, all items were used in the analyses | .80/ .85/ .89 | **Study 1**:  Kaiser–Meyer–Olkin measure: .828  Bartlett’s test of sphericity: χ²(df=21)=221.79, p<.001  Number of factors with eigenvalues > 1: 1  Used solution: 1  Explained variance: 45.79%  **Study 2 (T1):**  Kaiser–Meyer–Olkin measure: .818  Bartlett’s test of sphericity: χ²(df=21)=386.89, p<.001  Number of factors with eigenvalues > 1: 2  Explained variance: 68.43%  Used solution: 1 based on internal consistency and comparison with T2  **Study 2 (T2)**:  Kaiser–Meyer–Olkin measure: .859  Bartlett’s test of sphericity: χ²(df=21)=343.18, p<.001  Number of factors with eigenvalues > 1: 1  Explained variance: 60.54%  Used solution: 1 |
|  | I answer all my patients' questions sufficiently. |  |  |  |  |
|  | When I explain a treatment or procedure, I am sure that my explanation is completely correct. |  |  |  |  |
|  | I explain all examinations or procedures in a way that my patients understand. |  |  |  |  |
|  | I also involve the people accompanying the patients in decisions. |  |  |  |  |
|  | I listen to the patients' concerns and fears. |  |  |  |  |
|  | I consider how much prior knowledge a patient has and how much they can understand. |  |  |  |  |
|  | I can deal well with language barriers on the part of patients and accompanying persons in my everyday work. |  |  |  |  |
| **Outcome expectancies** |  | Three items used for the scale in the analyses | Shortened to three items | .76/ .73/ not used at T2 | **Study 1**:  Kaiser–Meyer–Olkin measure: .668  Bartlett’s test of sphericity: χ²(df=3)=101.76, p<.001  Number of factors with eigenvalues > 1: 1  Used solution: 1  Explained variance: 67.86%  **Study 2 (T1):**  Kaiser–Meyer–Olkin measure: .626  Bartlett’s test of sphericity: χ²(df=3)=96.80, p<.001  Number of factors with eigenvalues > 1: 1  Explained variance: 65.25%  Used solution: 1 |
| If I communicate well, … | ... the atmosphere in the team is good. |  |  |  |  |
|  | ... we take into account all important information in the course of treatment. |  |  |  |  |
|  | ... I need a lot of time in the beginning. | Poor item parameters, deleted from the analysis. | Not used for obstetric university sample |  |  |
|  | ... our patients are more satisfied. |  |  |  |  |
| **Action self-efficacy** |  | Both items used for the analyses | Changed to single-item scale | .77/ single-item scale at T1 and T2 in study 2 |  |
| I am sure that I can manage to communicate well… | … with my colleagues. |  | I am sure that I can manage to communicate well. |  |  |
|  | … with our patients. |  |  |  |  |
| **Coping self-efficacy** |  | All items were used in the analyses | Shortened to 4 items | .82/ .78/ .81 | **Study 1**:  Kaiser–Meyer–Olkin measure: .756  Bartlett’s test of sphericity: χ²(df=28)=377.50, p<.001  Number of factors with eigenvalues > 1: 2  Explained variance: 60.69%  Used solution: 1 based on internal consistency  **Study 2 (T1):**  Kaiser–Meyer–Olkin measure: .744  Bartlett’s test of sphericity: χ²(df=6)=159.53, p<.001  Number of factors with eigenvalues > 1: 1  Explained variance: 60.75%  Used solution: 1  **Study 2 (T2):**  Kaiser–Meyer–Olkin measure: .725  Bartlett’s test of sphericity: χ²(df=6)=105.76, p<.001  Number of factors with eigenvalues > 1: 1  Explained variance: 63.36%  Used solution: 1 |
| I am confident that I can communicate well even when … | … I am under time pressure. |  | Kept |  |  |
|  | ... my colleagues are under time pressure. |  | Omitted |  |  |
|  | ... there is little interest in good communication on the part of my colleagues. |  | Kept |  |  |
|  | ... communication with patients is already difficult. |  | Omitted |  |  |
|  | ... there are language barriers. |  | Kept |  |  |
|  | ... I am tired or exhausted. |  | Kept |  |  |
|  | ... I have not paid attention to communicating well for a long time. |  | Omitted |  |  |
|  | ... important information has not yet been communicated to me myself. |  | Omitted |  |  |
| **Intention** |  | All items used for the analyses | Changed to single-item scale | .88 | **Study 1**:  Kaiser–Meyer–Olkin measure: .723  Bartlett’s test of sphericity: χ²(df=3)=206.79, p<.001  Number of factors with eigenvalues > 1: 1  Explained variance: 80.62%  Used solution: 1 |
| I have resolved... | ... to always make sure that I communicate well with my colleagues. |  | I have resolved to always make sure that I communicate well. |  |  |
|  | ... to always pay attention to whether I communicate well with my patients. |  |  |  |  |
|  | ... to work regularly on my communication. |  |  |  |  |
| **Action planning** |  | All items used for the analyses | Changed to single-item scale | .93 | **Study 1**:  Kaiser–Meyer–Olkin measure: .726  Bartlett’s test of sphericity: χ²(df=3)=320.38, p<.001  Number of factors with eigenvalues > 1: 1  Explained variance: 87.57%  Used solution: 1 |
| I have already planned quite specifically, … | ... how I can communicate well. |  | Kept |  |  |
|  | ... on which occasion I can communicate well. |  | Omitted |  |  |
|  | ... with which persons I can communicate well. |  | Omitted |  |  |
| **Coping planning** |  | Both items used for the analyses | Changed to single-item scale | .81 |  |
| To be able to communicate well in difficult situations, I have already planned … | ... how I communicate best in such situations. |  | Even if something went wrong once, then I know how we can still communicate well. |  |  |
|  | ... how I can communicate well even if I haven't paid attention for a while. |  |  |  |  |
| **Perceived barriers** |  | All items used for the analyses | Shortened to 4 items, 3 items used for the analyses | .75/ .66 (not used at T2) | **Study 1**:  Kaiser–Meyer–Olkin measure: .681  Bartlett’s test of sphericity: χ²(df=15)=192.73, p<.001  Number of factors with eigenvalues > 1: 2  Explained variance: 63,56%  Used solution: 1 based on internal consistency  **Study 2 (T1):**  Kaiser–Meyer–Olkin measure: .636  Bartlett’s test of sphericity: χ²(df=3)=58.96, p<.001  Number of factors with eigenvalues > 1: 1  Explained variance: 59.73%  Used solution: 1 |
| For me, the following things interfere with good communication: | We have an emergency. |  | Kept and used |  |  |
|  | My colleagues have little interest in good communication. |  | Omitted |  |  |
|  | Relatives also want to be part of the communication. |  | Omitted |  |  |
|  | There are language barriers. |  | Kept and used |  |  |
|  | I am under time pressure. |  | Kept and used |  |  |
|  | There are precise guidelines for procedures and their documentation in everyday work. |  | Omitted due to poor item parameters |  |  |
| **Perceived patient safety** |  | All items used for the analyses | Shortened to 7 items (highest range in online survey) | .96/ .79/ .87 | **Study 1**:  Kaiser–Meyer–Olkin measure: .904  Bartlett’s test of sphericity: χ²(df=435)=2503.99, p<.001  Number of factors with eigenvalues > 1: 6  Explained variance: 70,22%  Used solution: 1 based on internal consistency  **Study 2 (T1)**:  Kaiser–Meyer–Olkin measure: .753  Bartlett’s test of sphericity: χ²(df=21)=262.78, p<.001  Number of factors with eigenvalues > 1: 2  Explained variance: 60.10%  Used solution: 1 based on internal consistency and comparison with T2  **Study 2 (T1)**:  Kaiser–Meyer–Olkin measure: .859  Bartlett’s test of sphericity: χ²(df=28)=283.34, p<.001  Number of factors with eigenvalues > 1: 1  Explained variance: 54.71%  Used solution: 1 |
| On a routine working day, I usually notice that... | ... colleagues have poorly washed or disinfected their hands. |  | Kept |  |  |
|  | ... colleagues or I myself were very stressed. |  | Omitted |  |  |
|  | ... there were conflicts among colleagues. |  | Kept |  |  |
|  | ... patients and their companions were poorly informed about examinations and treatment measures. |  | Kept |  |  |
|  | ... patients were mixed up. |  | Omitted |  |  |
|  | ... Patients have received the wrong or incorrectly dosed medication. |  | Omitted |  |  |
|  | ... diagnoses were made incorrectly. |  | Omitted |  |  |
|  | ... diagnoses were made prematurely or too late. |  | Omitted |  |  |
|  | ... patients have received the wrong treatment. |  | Omitted |  |  |
|  | ... colleagues or I myself have forgotten which measures (e.g. medication) were ordered for a patient. |  | Omitted |  |  |
|  | ... generally applicable duties of care were ignored. |  | Kept |  |  |
|  | ... colleagues forgot to pass on important information. |  | Kept |  |  |
|  | ... conversations were held "between door and handle". |  | Omitted |  |  |
|  | ... my opinions and suggestions were ignored. |  | Kept |  |  |
|  | ... important information (e.g. about allergies) was missing from a patient file. |  | Kept |  |  |
|  | ... technical terms were used with which the patients and their companions were overwhelmed. |  | Omitted |  |  |
|  | ... there were too few staff. |  | Omitted |  |  |
|  | ... communication with colleagues was poor. |  | Omitted |  |  |
|  | ... processes did not work smoothly. |  | Kept |  |  |
|  | ... colleagues or I myself were emotionally burdened. |  | Kept |  |  |
|  | ... colleagues or I myself were not familiar enough with technical equipment. |  | Kept |  |  |
|  | ... colleagues or I myself were poorly prepared for conversations with patients and their companions. |  | Kept |  |  |
|  | ... equipment was missing in the examination rooms. |  | Omitted |  |  |
|  | ... colleagues appeared unsafe. |  | Omitted |  |  |
|  | ... too few important or useful treatment options (e.g. medication, physiotherapy, etc.) were taken into account. |  | Kept |  |  |
|  | ... important information was missing from discharge reports. |  | Kept |  |  |
|  | ... patients were treated more like "numbers" and less like people. |  | Omitted |  |  |
|  | ... the cooperation between the hospital and external treatment providers did not work well. |  | Kept |  |  |
|  | ... I had difficulty communicating information due to language barriers on the part of patients and their companions. |  | Omitted |  |  |
|  | ... conflicts arose due to language barriers. |  | Kept |  |  |
| **Work satisfaction** |  | Not used | Only in the hospital sample, 3 items used for the analyses | .92/ .92 | **Study 2 (T1)**:  Kaiser–Meyer–Olkin measure: .757  Bartlett’s test of sphericity: χ²(df=3)=304.35, p<.001  Number of factors with eigenvalues > 1: 1  Explained variance: 86.61%  Used solution: 1  **Study 2 (T1)**:  Kaiser–Meyer–Olkin measure: .737  Bartlett’s test of sphericity: χ²(df=3)=180.34, p<.001  Number of factors with eigenvalues > 1: 1  Explained variance: 85.93%  Used solution: 1 |
|  | I am pretty happy with my current job. |  | Used |  |  |
|  | Most days I look forward to my work. |  | Used |  |  |
|  | I really enjoy my work. |  | Used |  |  |
|  | I consider my work to be rather unpleasant. |  | Omitted due to poor item parameters |  |  |
| **Feasibility** |  | Not used | Only in the hospital sample |  |  |
| **Overall conditions** |  |  | All items were used for the analysis | .75 (T2) |  |
|  | The size of the groups was appropriate for the training. |  |  |  |  |
|  | The groups were well mixed with the different professional groups. |  |  |  |  |
|  | The materials used helped me understand and implement the content. |  |  |  |  |
|  | The training was long enough to have a positive impact on communication. |  |  |  |  |
|  | The training was short enough that it did not become a burden for me. |  |  |  |  |
| **Trainers** |  |  | Used as single-item scale |  |  |
|  | The trainer was very knowledgeable about communication and patient safety. |  | Omitted due to poor item parameters |  |  |
|  | I had the impression that the trainer could still teach me something. |  | Used |  |  |
| **Training contents** |  |  | All items used for the analysis | .86 (T2) |  |
|  | The prerequisites for effective communication within the team have been sufficiently addressed. |  |  |  |  |
|  | The prerequisites for effective communication with patients and their companions were sufficiently addressed. |  |  |  |  |
|  | New communication strategies for stressful and emergency situations were taught. |  |  |  |  |
|  | I learned concrete application methods that help me communicate important information even under time constraints. |  |  |  |  |
|  | I learned techniques to ensure that the information I communicate is accurate. |  |  |  |  |
|  | Through the training, I have learned to pay even more attention to how my patients and their companions understand me. |  |  |  |  |
|  | After the training, it will be easier for me to also consider the environment in which I communicate. |  |  |  |  |
|  | I have learned in this training to be more attuned to the other members in the team. |  |  |  |  |
|  | I have learned in this training to adapt better to the patients and their companions. |  |  |  |  |
| **Benefit of the training** |  |  | All items used for the analysis | .90 (T2) |  |
| I believe that through the training.... | ... more important information will be passed on and heard by the team in the future. |  |  |  |  |
|  | ... patients and their companions will receive more important information in an understandable way in the future. |  |  |  |  |
|  | ... information in the team will be communicated more clearly in the future. |  |  |  |  |
|  | ... the course of treatment will be more comprehensible for patients and their companions. |  |  |  |  |
|  | ... everyone in the team will pay more attention to whether the environment and context are adequately taken into account in communication. |  |  |  |  |
|  | ... in the future, more attention will be paid to the concerns and needs of the other person. |  |  |  |  |
|  | ... patient satisfaction at our clinic will increase in the future. |  |  |  |  |
|  | ... patient safety at our clinic will increase in the future. |  |  |  |  |
| **Overall acceptability** |  |  | All items used for the analysis | .86 (T2) |  |
|  | Overall, the training met my expectations. |  |  |  |  |
|  | The training had a high general benefit for my everyday work. |  |  |  |  |
|  | I will be more attentive as a result of the training when my communication is not yet ideal. |  |  |  |  |

**Appendix 2. Overview over the communication training.**

| Duration in minutes | Content/ exercise | Description | Goal and transfer | HAPA construct (Schwarzer & Hamilton, 2020) | BCT taxonomy (Michie et al., 2013) |
| --- | --- | --- | --- | --- | --- |
| 30 | Introduction round | Introduction of the project and trainers, every participant chooses a picture card, introduces themselves and describes why this card represents a good childbirth/ communication. | Create an open atmosphere, illustrate differences in mindset | Setting the stage for change |  |
| 20 | Short film “Just a routine operation” | Participants are asked to look for communication deficits, moderated discussion on mistakes and possible solutions. Handout card “CRM” and “10-for-10”. | Enhance example-based learning, strengthenawareness of the role of team communication, illustrate communication failures, introduce the “10-for-10” rule and crew resource management (CRM) | Outcome expectancies, goals | Shaping knowledge [BCT4], Comparison of outcome [BCT9], Problem solving [BCT1.2] |
| 20 | Exercise “Tangram” | Two participants are sitting with their backs to each other and one participant gives instructions on how to place the Tangram tiles. The other participant arranges the tiles according to the instructions. In the end, the resulting form is compared to the template.  Moderated discussion on similar experiences in daily work. Handout card “Close-the-loop”. | Understanding that simply “correct” communication is not sufficient, train clear team communication (“closed-loop communication”) | Intention, self-efficacy, planning | Feedback and monitoring [BCT2], Associations [BCT7] |
| 15 | Active break | Participants are asked to find similarities, the person with the most similarities to others gets a reward (chocolate). | Positive experience and identity | Self-perception/ evaluation | Identity [BCT13] |
| 40 | Exercise “Empathy maps” | Each group creates an „empathy map“ for the other occupational group/ expectant mothers. Questions: What are their tasks/ feelings/ needs/ fears?  Point out common misunderstandings and similarities between groups. | Change of perspective, taking different roles, recognize outcomes of own actions | Intention, outcome experiences and self-efficacy | Natural consequences [BCT5], Goal setting (outcome) [BCT1.1], Commitment [BCT1.9], Social support [BCT3] |
| 35 | Exercise “Hand-over” | An unstructured hand-over is given to a first participant. This person repeats the hand-over to a second participant who was waiting outside the door. This participant gives a final recall.  Moderated discussion if and how participants structured their hand-over/ if information was lost or redundant. Handout card “Sufficiency”. | Introduce the team communication structure “ISBAR” (Introduction, Situation, Background, Assessment, Recommendation), sufficiency of information | Outcome expectations/ experiences,  Planning | Associations [BCT7], Behavioral practice/ rehearsal [BCT8.1], Antecedents [BCT12] |
| 10-15 | Exercise “Interpersonal adaption” | Participants are asked to inform a stressed expectant mother (trainer) about a concerning diagnosis. | Communication with different recipients, simplify language | Planning | Action planning [BCT1.4], Discrepancy between current behavior and goal [BCT1.6], Graded task [BCT8.7] |
| 10-15 | Exercise “Speaking-Up” | Presentation of a study on how few HCW spoke up in a simulated setting where a patient’s life was endangered. Discussion of a typical obstetric situation and roleplay to practice speaking-up. Handout card “Speaking-Up”. | Overcome hierarchies in teams and learn to voice patient safety concerns | Coping self-efficacy and planning | Action planning [BCT1.4], Commitment [BCT1.9], Covert learning [BCT16] |
| 10-15 | Discussion “Stop-Inject” | Participants are asked whether they have had medication errors before. Moderated discussion on how to use communication to avoid medication errors. Handout card “Stop-Inject”. | Avoid medication errors through safe communication | Planning, self-efficacy | Action planning [BCT1.4], Behavioral practice/ rehearsal [BCT8.1], Self-belief [BCT15] |
| 5 | Wrap-up | Trainers summarize the training contents. | Recap |  |  |
| 30 | Behavior planning intervention | Participants are given the behavior planning intervention sheet. They are asked to identify a key component/ aspect of communication they want to work on independently and fill in the sheet (c.f. appendix 2). | Identify goals for individual learning after the training , transfer to everyday work life, maintenance | Action and coping planning, maintenance | Goal setting (behavior) [BCT1.1], Action planning [BCT1.4], Habit formation [BCT8.3] |
| 10 | Short evaluation | Participants are handed the feasibility questionnaire; a short feedback round is completed. |  |  |  |

**Appendix 3. Planning intervention.**

(BCT taxonomy Michie et al., 2013 – Action Planning BCT1.4, Review behavior goal(s) BCT1.5, Behavioral contract BCT1.8 and Commitment BCT1.9).

**Planning your Communication in Daily Work Life after this Training**

What are your plans for the near future? Please fill in one, two or three concrete **communication activities** in the boxes that you specifically want to do (well or better than before).

| Which communication activities do you want to do (better than before)?  (Please put one activity in each box, e.g. “In the future, I’ll ask the expectant mothers if they understood what I explained.”) | | |
| --- | --- | --- |
| Communication activity a) | Communication activity b) | Communication activity c)^[[1]](#footnote-1)^ |

Now we want to **plan these activities** more precisely. Please think of the „communication activity a“ and answer the questions 1 to 8. When you finish with activity a), please answer the questions again for the communication activities that you have entered under b) and c).

| 1. **How** often do you want to carry out the activities?  (please fill in the frequency per week or every day) | | |
| --- | --- | --- |
| Communication activity a) | Communication activity b) | Communication activity c) |
| 2. **How long** do you plan for each activity?  (please fill in the length of each communication activity) | | |
| Communication activity a) | Communication activity b) | Communication activity c) |
| 3. **Where** do you want to carry out the activities?  (please fill in the respective location) | | |
| Communication activity a) | Communication activity b) | Communication activity c) |
| 4. **When** do you want to realize the activities?  (please fill in typical situations e.g. „on the first encounter“ or „after greeting each other“) | | |
| Communication activity a) | Communication activity b) | Communication activity c) |
| 5. **Who** do you want to carry out the activities with? | | |
| Communication activity a) | Communication activity b) | Communication activity c) |
| 6. **What tools** do you need in order to carry out your activities?  (please indicate which tools you need e.g., paper and pen, extra space, notepad, etc.) | | |
| Communication activity a) | Communication activity b) | Communication activity c) |
| 7. When do you want to **start putting your plans into action** (the ones outlined above)?  (please indicate when you want to start) | | |
| Communication activity a) | Communication activity b) | Communication activity c) |
| 8. **What could prevent or make it difficult** for you to carry out your planned communication activities? (please fill in at least one difficulty per communication activity) | | |
| Communication activity a) | Communication activity b) | Communication activity c) |

Please think about **how you can carry out your planned communication activities despite these difficulties**. Try to find at least one possibility in order to communicate well despite the difficulties.

| 9. How would you communicate well in spite of the difficulties? | | |
| --- | --- | --- |
| Communication activity a) | Communication activity b) | Communication activity c) |

Please take **another look** at your plans.

| 10. What do you think about your plans? |
| --- |
|  |

*Congratulations on finishing these plans!*

*Good luck practicing your communication activities and enjoy better communication!*

**Appendix 4. Microteachings.**

| Topic | Content/ exercise |
| --- | --- |
| Speaking-Up | Two microteaching units.  *First unit:* A multiple choice question followed by a study manuscript and a pocket card to print out.  Question:  A consultant asks a nurse or a resident to prepare a potentially lethal medication dosage. How many staff members would speak up?  a) All staff members.  b) 7 out of 10.  c) About half of them.  d) about one third of them.  e) Just 1 out of 10.  Study manuscript:  St Pierre, M., Scholler, A., Strembski, D., & Breuer, G. (2012). Do residents and nurses communicate safety relevant concerns?: simulation study on the influence of the authority gradient. Der Anaesthesist, 61(10), 857-866. <https://doi.org/10.1007/s00101-012-2086-1>  *Second unit:* A multiple choice question followed by a link to the Swiss patient safety agency.  Question:  Which methods do you know for speaking-up?  a) To loudly criticize the consultant’s decision in attendance of the pregnant woman.  b) Using codewords (e.g., asking to speak about Mrs Jacobs).  c) Using a traffic light metaphor (e.g., “I feel yellow”).  d) Saying: “Are you crazy?? That’s dangerous!”  e) Saying: “I need to talk to you outside about another patient urgently.”  Link:  <https://www.patientensicherheit.ch/speak-up/> |
| Sufficiency | One microteaching unit with a multiple choice question followed by a graph illustrating the ideal amount of information.  Question:  Why is it important to mind a sufficient quantity of information?  a) The attention of my counterpart is limited to a certain amount of information.  b) More information is always better.  c) If I don’t give enough information, my counterpart will not be able to assess the situation adequately. |
| ISBAR | One microteaching unit with a multiple choice question followed by an article and a pocket card.  Question:  What does the acronym ISBAR stand for?  a) Information, safety, briefing, attention, regard.  b) Introduction, situation, background, assessment, recommendation.  c) Induction, suction cup, birth process, (medical) assistant, recall.  Link to the article:  <https://www.aerzteblatt.de/archiv/197286/Kommunikation-Absprachen-klar-strukturieren> |
| Medication errors | One microteaching unit with a multiple choice question followed by a study manuscript and a pocket card.  Question:  What are elements of the 5-R-rule?  a) Right patient.  b) Right medication.  c) Right application.  d) Right timepoint.  e) Right indication.  f) Right dosage.  g) Right room number.  Study manuscript:  Sutherland, A., Canobbio, M., Clarke, J., Randall, M., Skelland, T., & Weston, E. (2020). Incidence and prevalence of intravenous medication errors in the UK: a systematic review. European Journal of Hospital Pharmacy, 27(1), 3-8. <https://doi.org/10.1136/ejhpharm-2018-001624> |
| Core competencies | One microteaching unit with a multiple choice question followed by a pocket card.  Question:  Key communication competencies include:  a) Safety.  b) Clarity.  c) Balance.  d) Correctness.  e) Interpersonal adaption.  f) Speed.  g) Sufficiency. |
| Crew Resource Management | One microteaching unit with a multiple choice question followed by a pocket card.  Question:  “Be a leader or a good team member showing persistence.” What is meant by this principle of CRM?  a) Try to be the leader, no matter what.  b) Take the lead if you’re fit to do so and no one else takes it.  c) As a good team member, you only have to carry out instructions, not think about them.  d) As a good team member, you voice justified concerns emphatically, if necessary.  e) “Showing persistence” means “until you get your way”. |
| Close-the-loop | One microteaching unit with a multiple choice question followed by a pocket card.  Question:  What does closed-loop communication mean?  a) Directly addressing someone, if possible using the name of that person.  b) Repeating instructions that you’ve been given so that the instructor knows you understood them correctly.  c) Ask as many questions as possible.  d) Report back to the instructor that you finished the assigned task.  e) Wait for the confirmation of the instructor that they perceived you fulfilled the task.  f) Eye contact is enough of a confirmation. |
| (Interpersonal) adaption | One microteaching unit with a multiple choice question followed by a pocket card.  Question:  In which of the following examples did (interpersonal) adaption work?  a) Giving the resident new information about a patient before he sees another patient.  b) Assigning a female doctor to a Muslim female patient.  c) Voicing a suspected diagnosis at short rounds.  d) Telling a colleague about a mistake you noticed in a private conversation instead of calling it out in front of everyone. |
| 10-for-10 | One microteaching unit with a multiple choice question followed by a study manuscript and a pocket card.  Question:  Which statements about “10-for-10” are correct?  a) Only the consultant can initiate a “10-for-10”.  b) A “10-for-10” can help to recognize fixation errors.  c) Everyone in a team can initiate a “10-for-10”.  d) During a “10-for-10”, all manual tasks should be halted.  e) During a “10-for-10”, every team member is allowed to voice recommendations.  f) A “10-for-10” should only be initiated if a patient’s condition is critical. |

1. In the planning intervention, all HCW who took part in the training were asked to reconsider what they had learned and choose up to three communication components that they wanted to improve in their working life after the training. They were asked how they would improve these communication components by specifically planning the transfer into their working life. Choosing and planning certain communication behaviors was implemented in the training to overcome the intention-behavior gap and facilitate the transfer of what was learned into actual behavior after the training. The rationale to choose up to 3 components was to offer HCW to identify their individual strengths as well as weaknesses and to work on them specifically. Three components were considered the limit of what could be improved in routine work life without the danger of discouragement if high goals were not met. [↑](#footnote-ref-1)
